# Surgical treatment of a rare case of atypical polypoid adenomyoma of the uterus in a menopausal patient: a case report

**DOI:** 10.11604/pamj.2023.44.118.39256

**Published:** 2023-03-07

**Authors:** Efthymia Ioannis Thanasa, Anna Ioannis Thanasa, Ioannis Emmanouel Paraoulakis, Evangelos Konstantinos Kamaretsos, Vasiliki Anastasios Grapsidi, Gerasimos Periklis Kontogeorgis, Evangelos-Ektoras Athanasios Gerokostas, Athanasios Nikolaos Chasiotis, Vasileios Christos Kontochristos, Ioannis Konstantinos Thanasas

**Affiliations:** 1 Department of Health Sciences, Medical School, Aristotle University of Thessaloniki, Thessaloniki, Greece,; 2 Department of Obstetrics and Gynecology of General Hospital in Trikala, Trikala, Greece,; 3 Department of Obstetrics and Gynecology of General Hospital in Limassol, Limassol, Cyprus

**Keywords:** Atypical polypoid adenomyoma, ultrasound, curettage, treatment, case report

## Abstract

Atypical polypoid adenomyoma is a rare benign tumor of the uterus that usually affects women of reproductive age and has an increased risk of progression into endometrial cancer. The pathogenetic mechanism has not been completely clarified. Due to the rarity of the tumor, current experience regarding the diagnostic and therapeutic approach is limited. For menopausal patients, hysterectomy seems to be the main treatment option. Our case concerns an asymptomatic menopausal patient with a vaginal delivery in her obstetric history and no hereditary history of gynecological cancer who came to the outpatient clinic for a gynecological examination. Transvaginal ultrasound revealed the presence of a large, round solid mass with increased vascularity within the endometrial cavity. A diagnostic dilation and curettage of the endometrium was performed. Histological examination of the endometrial biopsy showed an atypical polypoid adenomyoma, and it was decided to perform a total abdominal hysterectomy with bilateral adnexectomy. Histological examination of the surgical specimen of the uterus revealed no residual disease, no coexisting foci of atypical endometrial hyperplasia or endometrial cancer. The postoperative course was uneventful. The patient remains to this day under regular follow-up. The present case report highlights the significant difficulties involved in the preoperative diagnosis of atypical polypoid adenomyoma of the uterus and the difficult differential diagnosis from atypical endometrial hyperplasia and endometrial cancer, particularly in menopausal patients. At the same time, it is pointed out that despite its rarity, the early diagnosis of atypical polypoid adenomyoma, especially in young women, must be the main concern of the modern gynaecologist, in order to design the optimal treatment aimed at preserving fertility while avoiding the risk of recurrence of damage or malignant progression into endometrial cancer.

## Introduction

In the current World Health Organization classification of mixed epithelial and mesenchymal tumors of the uterus, among carcinosarcoma, adenosarcoma, adenofibroma, and adenomyoma, atypical polypoid adenomyoma of the uterus is included [1]. Atypical polypoid adenomyoma or atypical polypoid adenomyofibroma, as it is otherwise referred to in the literature, is an uncommon benign tumor of the uterine corpus, the development of which involves a compound of cells of epithelial and mesenchymal origin [2]. To date, less than 250 cases have been reported in the world literature [3]. In this study, after describing the case, a brief literature review of this uncommon uterine tumor is attempted, pointing out that, due to its rarity, the current experience regarding the diagnostic and therapeutic approach is limited.

## Patient and observation

**Patient information:** a 53-year-old menopausal patient came to the gynecology outpatient clinic to undergo a routine gynecological examination. The patient had a vaginal delivery in her obstetric history and had a personal medical history of arterial hypertension, well-regulated with medication. Since the last preventive gynecological examination three years ago, no pathological findings were reported. The patient had no family history of gynecological cancer. From her personal medical history, there was no mention of menstrual disorder during her reproductive years, nor of abnormal vaginal bleeding during the last three years that the patient was in menopause. In addition, the patient has never received hormonal contraception, nor hormone replacement therapy in the last three years.

**Clinical findings:** the patient was asymptomatic. She reported no feeling of vaginal fullness or heaviness, nor did she report episodes of colicky or other type of lower abdominal pain. During the gynecological examination of the patient, a well-epithelized cervix was found, without the presence of macroscopic lesions. Cytological examination of cells taken from the ectocervix and endocervix was negative for malignancy.

**Figure 1:**
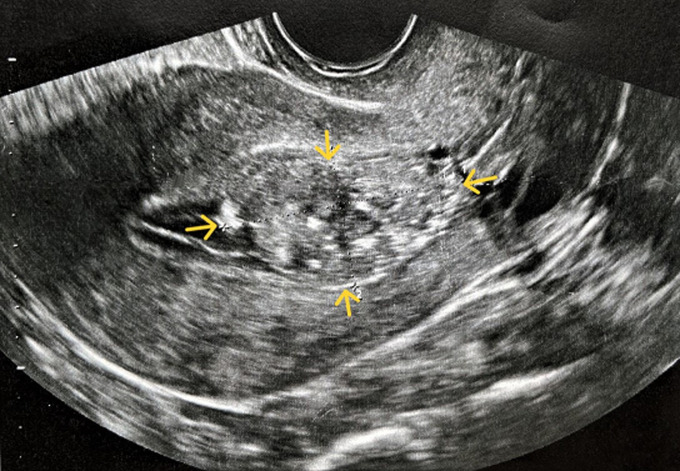
transvaginal ultrasound imaging of an atypical polypoid adenomyoma of the uterus; a solid, round-shaped, well-defined mass (yellow arrows) within the endometrial cavity is easily visualized

**Figure 2:**
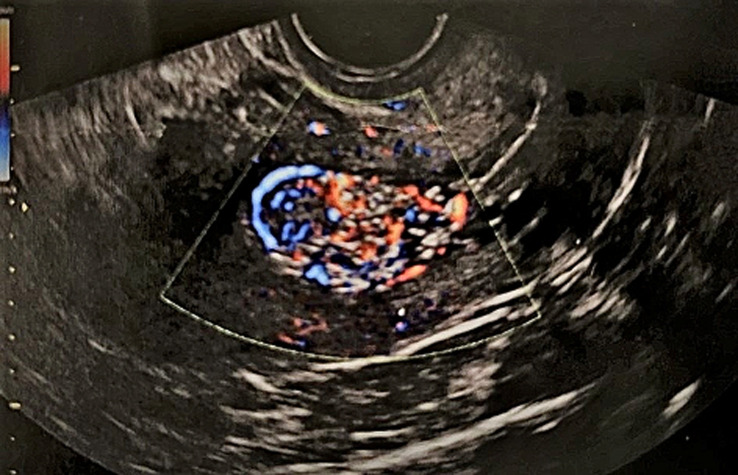
transvaginal Doppler ultrasound imaging of an atypical polypoid adenomyoma of the uterus; the imaging of increased vascularity of the endometrial mass

**Diagnostic assessment:** with the transvaginal ultrasound, the presence of a large round solid mass in the endometrial cavity, measuring 39x25mm, was established (Figure 1). Assessment of tumor blood flow using Doppler ultrasound showed increased vascularity (Figure 2). The size of the uterus was normal for the patient's age, and no pathological findings from the ovaries were visualized. On the admission of the patient to our clinic, from the laboratory analysis, it was found: Ht 40.1%, Hb 13.3 gr/dl, PLT 233x103/ml, U 35 mg/dl, Cr 0.7 mg/dl, Na 138 mEq/lt, K 4.1 mEq/lt. Inflammatory markers and urinalysis were within normal range. Tumor markers (CA125, CA15-3, CA19-9) were negative.

**Figure 3:**
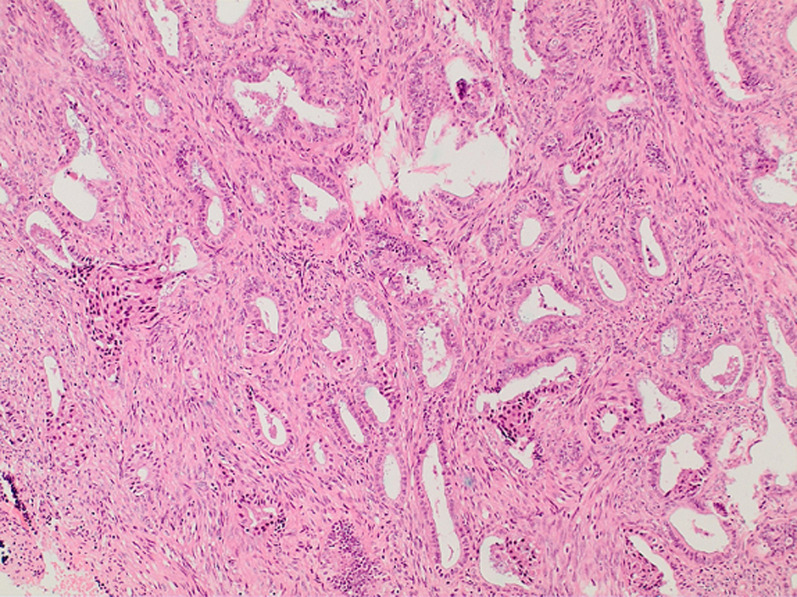
histological image of an atypical polypoid adenomyoma of the uterus; the presence of a myofibromatous stroma without cytological atypia and endometrial glands of variable size with layering of nuclei and unequal size as on mild atypiais characteristic

**Figure 4:**
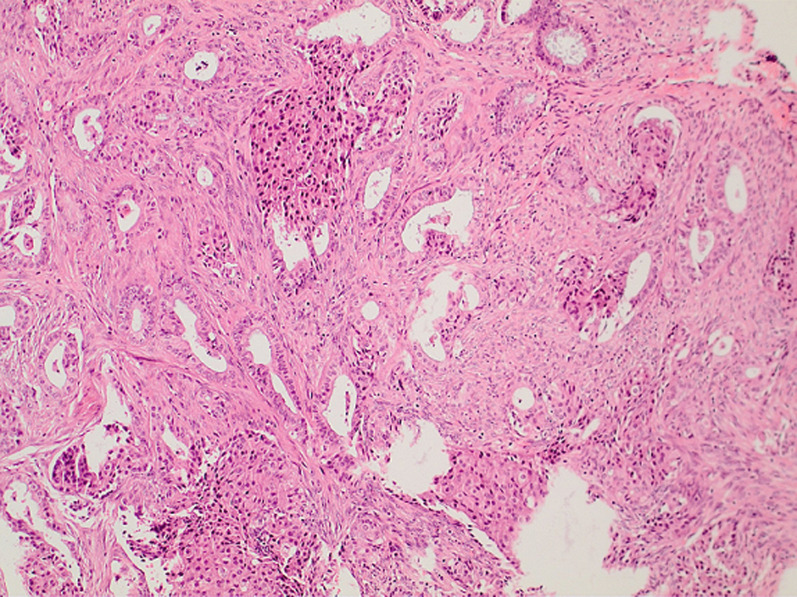
histological image of an atypical polypoid adenomyoma of the uterus; focal squamous metaplasia of endometrial glands is evident

**Therapeutic intervention:** after detailed consultation with the patient and her relatives about the therapeutic approach to the disease, a diagnostic dilation and curettage was performed. Histological examination of the endometrial sampling showed an atypical polypoid adenomyoma with the presence of a myofibromatous stroma without cytological atypia and endometrial glands of variable size with focal squamous metaplasia and layering of nuclei with unequal size as on mild atypia (Figure 3, Figure 4). It was then decided to perform a total abdominal hysterectomy with bilateral adnexectomy. The magnetic resonance imaging performed as part of the preoperative evaluation, before performing the hysterectomy, did not show pathological findings from the uterus and parametrium, nor abnormally enlarged lymph nodes. No immediate complications related to the surgery were reported. Histological examination of the surgical specimen of the uterus revealed no residual disease, no coexisting foci of endometrial hyperplasia with atypia or endometrial cancer.

**Follow-up and outcomes:** after a smooth postoperative course, the patient was discharged from our clinic on the fourth postoperative day. The patient is attended at regular intervals in the gynecology outpatient clinic. Six months after performing the total hysterectomy, the findings from the clinical and cytological examination of the vaginal vault remain normal.

**Patient perspective:** the patient was satisfied with the treatment she received.

**Informed consent:** this was sought and obtained from the patient. Anonymity was maintained for confidentiality.

## Discussion

Atypical polypoid adenomyoma was first described by Mazur in 1981 [4]. It is a rare benign polypoid tumor of the uterus that usually affects nulliparous women of reproductive age and is associated with infertility [5]. Its occurrence in menopause, as in our patient, is extremely rare. Atypical polypoid adenomyoma is usually located in the lower part of the uterus or in the endocervix or in the endometrial cavity (our case) and may coexist with atypical endometrial hyperplasia and/or endometrial adenocarcinoma [6]. Recently, a unique case of localization of an atypical polypoid adenomyoma in the vaginal vault, after performing a total abdominal hysterectomy to treat endometrial hyperplasia with atypia, has been described in the world literature [2]. The exact pathogenetic mechanism has not been completely clarified. Many studies to date demonstrate a possible association between atypical polypoid adenomyoma of the uterus and estrogen-related factors. More specifically, factors such as obesity, diabetes mellitus, prolonged estrogen stimulation and hormone replacement therapy are estimated to be related to the pathogenesis of this rare pathological entity [7]. Also, ovarian dysfunction caused by hyperprolactinemia is thought to be involved in the pathogenesis of atypical polypoid adenomyoma of the uterus [8]. More recently in 2015, Němejcová et al. analyzing the results of a comprehensive immunohistochemical and molecular analysis of a case series of the disease concluded that, given the association with atypical endometrial hyperplasia and endometrioid adenocarcinoma and common immuno histochemical and molecular features, atypical polypoid adenomyoma of the uterus could conceptually be better considered as analogous to a localized type of atypical hyperplasia [9].

Due to the rarity of the tumor, the diagnostic approach is limited. The preoperative diagnosis of atypical polypoid adenomyoma of the uterus is a challenge in modern surgical clinical practice. The main clinical feature of atypical polypoid adenomyoma of the uterus is abnormal vaginal bleeding. Abnormal vaginal bleeding usually takes the form of hypermenorrhea (almost in all cases) or may refer to irregular menstruation or abnormal bleeding other than menstrual [6]. In addition, anemia and infertility are included in the main clinical features characterizing atypical polypoid adenomyoma of the uterus [10]. It is remarkable that our patient did not have any predisposing factor or clinical symptom: our patient with no history of infertility was not nulliparous, was not obese (BMI = 25.31), did not have anemia, never received hormonal contraception, nor did she receive hormone replacement therapy in menopause. In addition, the menstrual cycle during reproductive age was regular, and she never had vaginal bleeding during the recent years she was in menopause.

Imaging, including ultrasonography, Computed Tomography, and Magnetic Resonance Imaging may be used as part of the preoperative evaluation for preliminary assessment of the condition of uterus and parametrium. Transvaginal ultrasound and Doppler ultrasound imaging can reveal heterogeneous endometrial thickening, with polypoid adenomyomas depicted as solid, well-defined endometrial masses with cystic areas and changes in blood flow [11]. In our patient, the ultrasound imaging within the endometrial cavity is characteristic of a large (maximum diameter of approximately 40 mm) well-defined round solid mass with increased vascularity (Figure 1, Figure 2). In any case, however, as in our patient, the differential diagnosis of atypical polypoid adenomyoma of the uterus from endometrial cancer, atypical endometrial hyperplasia, endometrial polyps, adenomyosis and malignant mixed Mullerian tumors is deemed necessary [12]. In addition, hysteroscopy which should be indicated mainly in cases of infertility with abnormal uterine bleeding and pathological ultrasound findings can help in the diagnostic approach of atypical polypoid adenomyoma of the uterus. Despite the fact that the method offers direct endoscopic visualization of the lesion, the absence of a characteristic appearance of the tumor during hysteroscopy often causes confusion and misdiagnosis with endometrial polyps or submucosal fibroids. Nevertheless, the presence of an endometrial mass with a diameter > 1 cm, in combination with the presence of many and wide blood vessels on its surface, advocates for the diagnosis of atypical polypoid adenomyoma of the uterus. Finally, three-dimensional sonohysterography has been reported to be a good method of screening to address patients to hysteroscopic confirmation, especially in those with suspected polyps, myoma, mucus accumulation, and Mullerian abnormalities [13].

Similarly, it is currently estimated that even with histological examination, the differential diagnosis of atypical polypoid adenomyoma from my invasive endometrial adenocarcinoma remains difficult and remains to be clarified, as the glandular component presents endometrioid features with irregular architecture and cytological atypia. Histologically, atypical polypoid adenomyoma is a biphasic lesion of glandular and squamous cell proliferation, composed of atypical endometrial glands with squamous molecular differentiation in a background of abundant fibromuscular stroma [9]. Microscopically, the characteristic absence of cytoarchitectonic atypia of the surface epithelium in atypical polypoid adenomyoma contributes significantly to its differentiation from atypical endometrial hyperplasia and well-differentiated endometrial adenocarcinoma [4]. In our menopausal patient, the presence of endometrial glands of variable size with focal squamous metaplasia and layering of nuclei with unequal size as on mild atypia posed difficulties regarding the differentiation of the tumor from endometrial cancer. In addition, a recent study showed that distinct features of the fibromuscular stroma can help in the differential diagnosis between atypical polypoid adenomyoma of the uterus and endometrial carcinoma [14].

As a consequence of the rarity of atypical polypoid adenomyoma and the lack of prospective clinical trials, to date, no therapeutic protocols and guidelines have been established for the optimal management of these patients. In those cases where the disease concerns women of reproductive age who wish future childbearing, the indicated treatment option must be accompanied by the preservation of fertility. Progestin-based hormone therapy, hysteroscopic transcervical resection, cervical dilation and curettage, progestin-based hormone therapy combined with cervical dilation and curettage, and progestin-based hormone therapy combined with hysteroscopic transcervical resection are main conservative treatments for patients with atypical polypoid adenomyoma of the uterus who have not completed their family and wish to achieve a future pregnancy. The main disadvantages of conservative treatment approaches of the disease include the increased risk of recurrence of the lesion and the necessity of lifelong regular follow-up of the patient, with a real risk of malignant transformation of the disease [15,16]. Four-step hysteroscopic transcervical resection is now considered to be the first-line conservative treatment option. Hysteroscopic transcervical resection appears to be significantly superior to other conservative treatment approaches in terms of efficacy and safety [4,17].

Hysterectomy is the main treatment option in menopausal patients and in reproductive age patients who do not wish to preserve fertility [18]. In our patient, taking into account 1) the high rate of recurrence of the disease in the case of conservative treatment, 2) the relatively increased probability of coexistence of atypical endometrial hyperplasia and endometrioid carcinoma, 3) the patient's age and 4) the absence of patient´s desire to preserve fertility, it was decided performing total abdominal hysterectomy with adnexectomy, as the most appropriate and safest therapeutic approach, accompanied by regular follow-up of the patient with clinical and cytological examination of the vaginal vault. Follow-up of patients after conservative treatment is based on transvaginal ultrasound and the endometrial sampling every 3 months for the first 2 years, every 4 to 6 months for another 3 years, and once a year thereafter [17]. The recurrence rate of atypical polypoid adenomyoma of the uterus is estimated to be high, ranging from 28.9% to 35.1% [12,17]. A multicenter study showed that the rate of malignant transformation of the lesion is much higher than that of endometrial polyps and is estimated to be up to 0.8% of cases [19].

## Conclusion

Atypical polypoid adenomyoma of the uterus is extremely rare in menopause. In all cases, accurate preoperative diagnosis is a challenge in Gynecology today. Careful histological diagnosis and differentiation of atypical polypoid adenomyoma from endometrial cancer is of great value in the correct selection of the most appropriate therapeutic management of the disease, in order to ensure the best health of these patients. Preservation of the uterus in women of reproductive age is considered necessary in many cases, while hysterectomy seems to be the optimal treatment in menopausal patients.
